# Dysregulation of prostaglandins, leukotrienes and lipoxin A_4_ in bronchiectasis

**DOI:** 10.1136/thoraxjnl-2020-216475

**Published:** 2021-11-17

**Authors:** Pallavi Bedi, Kerstin Ziegler, Phil D Whitfield, Donald Davidson, Adriano Giorgio Rossi, Adam T Hill

**Affiliations:** 1 MRC Centre for Inflammation Research, University of Edinburgh, Edinburgh, UK; 2 Department of Lipidomics, University of the Highlands and Islands, Inverness, UK; 3 Department of Lipidomics, University of Glasgow, Glasgow, UK; 4 Respiratory Medicine, MRC Centre for Inflammation Research, University of Edinburgh, Edinburgh, UK

**Keywords:** bronchiectasis, neutrophil biology

## Abstract

**Introduction:**

Bronchiectasis is characterised by excessive neutrophilic inflammation. Lipid mediators such as prostaglandins and leukotrienes have crucial roles in the inflammatory response. Further characterisation of these lipids and understanding the interplay of anti-inflammatory and proinflammatory lipid mediators could lead to the development of novel anti-inflammatory therapies for bronchiectasis.

**Aim:**

The aim of our study was to characterise the lipids obtained from serum and airways in patients with bronchiectasis in the stable state.

**Methods:**

Six healthy volunteers, 10 patients with mild bronchiectasis, 15 with moderate bronchiectasis and 9 with severe bronchiectasis were recruited. All participants had 60 mL of blood taken and underwent a bronchoscopy while in the stable state. Lipidomics was done on serum and bronchoalveolar lavage fluid (BALF).

**Results:**

In the stable state, in serum there were significantly higher levels of prostaglandin E_2_ (PGE_2_), 15-hydroxyeicosatetranoic acid (15-HETE) and leukotriene B_4_ (LTB_4_) in patients with moderate–severe disease compared with healthy volunteers. There was a significantly lower level of lipoxin A_4_ (LXA_4_) in severe bronchiectasis.

In BALF, there were significantly higher levels of PGE_2_, 5-HETE, 15-HETE, 9-hydroxyoctadecadienoic acid and LTB_4_ in moderate–severe patients compared with healthy volunteers.

In the stable state, there was a negative correlation of PGE_2_ and LTB_4_ with % predicted forced expiratory volume in 1 s and a positive correlation with antibiotic courses.

LXA_4_ improved blood and airway neutrophil phagocytosis and bacterial killing in patients with bronchiectasis. Additionally LXA_4_ reduced neutrophil activation and degranulation.

**Conclusion:**

There is a dysregulation of lipid mediators in bronchiectasis with excess proinflammatory lipids. LXA_4_ improves the function of reprogrammed neutrophils. The therapeutic efficacy of LXA_4_ in bronchiectasis warrants further studies.

**Key messages**

**What is the key question?**

- Is there a dysregulation of lipids in bronchiectasis that leads to unremitting and chronic inflammation?

**What is the bottom line?**

- Ex vivo, lipoxin A_4_ can improve the function of reprogrammed blood and airway neutrophils in bronchiectasis.

**Why read on?**

- This is the first study assessing the role of lipids in bronchiectasis in depth. Here we have demonstrated that dysregulation of the proinflammatory and anti-inflammatory lipids in bronchiectasis contribute to failure of resolution of inflammation. Targeting the lipid pathways to initiate the resolution process in bronchiectasis may lead to development of novel non-antibiotic therapy in the stable state.

## Introduction

Excessive inflammation is widely accepted to be a unifying component in many chronic diseases, including bronchiectasis, vascular diseases, metabolic syndrome and neurological diseases, and thus is a public health concern. Understanding endogenous control points within the inflammatory response could potentially provide us with new perspectives on disease pathogenesis and treatment approaches. Break in the barrier, trauma and microbial invasion encourage the host to clear microbial pathogens, remodel and regenerate tissue. The acute inflammatory response is protective and usually self-limiting. Oxidative lipid products including, notably, the products of unsaturated lipids are increasingly being recognised as important contributors to chronic inflammatory diseases.^1 2^ Eicosanoids^3 4^ produced from the n-6 polyunsaturated fatty acid, arachidonic acid (20:4), as well as many cytokines and chemokines,^5 6^ have crucial roles in the initial response. Interactions among prostaglandins, leukotrienes and proinflammatory cytokines amplify inflammation, the signs and effects of which can be reduced by pharmacological inhibition and receptor antagonists.^3–5^ However, given that excessive inflammation contributes to several widely occurring diseases, improvements are required in treatment and in our understanding of the mechanisms involved. Eicosanoids also include lipoxins that possess potent anti-inflammatory properties.^7^ Further, n-3 fatty acids (eicosapentaenoic acid 20:5, and docosahexaenoic acid, 22:6) are precursors for proresolving mediators such as resolvins and maresins that limit the duration and magnitude of inflammatory responses.^8^ Recent advances have facilitated more detailed profiling of lipid mediators in serum and at the sites of inflammation.^9–11^ Characterisation of these lipids could thereby lead to the development of novel anti-inflammatory therapies for chronic inflammatory conditions.

Bronchiectasis is characterised by recurrent cough, daily sputum production and recurrent chest infections. There is excessive neutrophilic inflammation, but the driver for this unremitting inflammation is not known. We hypothesise that there is a failure of resolution of inflammation in bronchiectasis. Recently, we have been investigating the lipid pathway to establish if there is a dysregulation of the lipids in bronchiectasis contributing to the chronic inflammation. While this could be contributing to the persistent chronic inflammatory state in bronchiectasis, there is currently no data in the literature to indicate the interplay of lipid mediators in bronchiectasis.

The aim of our study was to characterise the lipids obtained from blood and airway samples in patients with bronchiectasis in the stable state and to assess the efficacy of lipoxin A_4_ (LXA_4_) on neutrophil function.

## Methods

Six healthy volunteers (partners of the patients recruited in the study with no background medical conditions and not currently on medication), 10 patients with mild bronchiectasis, 15 with moderate and 9 with severe bronchiectasis were recruited. All participants had 60 mL of blood taken and underwent a bronchoscopy. Two segments of the lungs were washed out in patients with bronchiectasis, an area affected by bronchiectasis and an area unaffected by bronchiectasis, predetermined by CT scan of chest. This led to patients acting as their own internal control. Lipidomics was done on blood and bronchoalveolar samples were obtained.

### Bronchiectasis severity

The severity of bronchiectasis was calculated using the Bronchiectasis Severity Index (BSI).^12^ The BSI is a risk stratification tool for morbidity and mortality in bronchiectasis. The minimum score is 0 and the maximum score is 26. A score between 0 and 4 indicates mild disease; 5–8 indicates moderate disease; and a score of ≥9 indicate severe disease. The BSI was calculated in all patients with bronchiectasis taking part in the study.

#### Inclusion criteria

Inclusion criteria include idiopathic or postinfective bronchiectasis, age >18 years and no infective exacerbation of bronchiectasis for at least 4 weeks prior to giving serum/bronchoscopy.

#### Exclusion criteria

Patients on statin, aspirin, inhaled corticosteroids and long-term macrolides were excluded.

### Bronchoscopy

All participants underwent a bronchoscopy. Participants were sedated with midazolam±fentanyl. Bronchoalveolar lavage (BAL) and brushings were obtained. For patients with bronchiectasis, BAL was done in an area *affected* by bronchiectasis and in an area *unaffected* by bronchiectasis as identified on CT scans done prior to bronchoscopy.

### Liquid chromatography–tandem mass spectrometry (LC-MS/MS) analysis of eicosanoids

Quantification of eicosanoids and related lipid mediators in patient bronchoalveolar lavage fluid (BALF) and serum samples was performed using LC-MS/MS^13^ (see online supplemental file, online supplemental figure 1 and online supplemental table T1).

10.1136/thoraxjnl-2020-216475.supp1 Supplementary data

**Figure 1:**
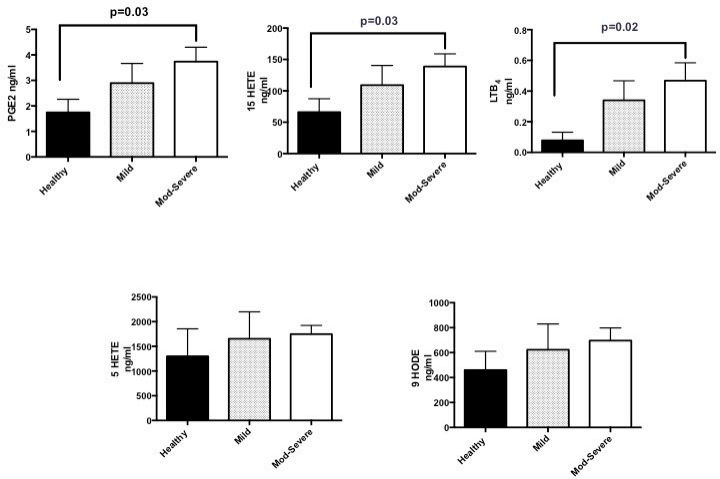
Significantly higher levels of PGE_2_, 15-HETE and LTB_4_ detected in patients with moderate–severe disease compared with healthy volunteers; p=0.03, p=0.03 and p=0.02, respectively. higher levels of 5-HETE and 9-HODE detected in patients with more moderate–severe disease compared with healthy volunteers, but not statistically significant. Lipidomics were obtained by mass spectrometry and liquid chromatography. Pooled data presented as mean±SEM. One-way analysis of variance used for comparisons. Healthy=6 volunteers, mild=9 patients, moderate–severe=15 patients. 5-HETE, 5-hydroxyeicosatetranoic acid; 15-HETE, 15-hydroxyeicosatetranoic acid; 9-HODE, 9-hydroxyoctadecadienoic acid; LTB_4_, leukotriene B_4_; PGE_2_, prostaglandin E_2_.

### LXA_4_ detection by ELISA

Serum LXA_4_ was measured as per manufacturer’s instruction (Neogen).

LXA_4_ was the lipid of choice for all the experiments outlined in this article as pilot data (data not shown in this paper) using sputum and blood from bronchiectasis exacerbations showed that LXA_4_ was the only lipid that improved after treatment with antibiotics.

### Isolation of blood and airway neutrophils

Freshly drawn blood was collected into 3.8% sodium citrate, and granulocytes were subsequently isolated by dextran sedimentation and discontinuous Percoll gradient.^14^ Sputum and BAL were washed and treated with sputolysin, and airway neutrophils were isolated. Anti-CD16 antibodies (Abcam) were used to identify neutrophils by flow cytometry.

### LXA_4_ function on reprogrammed bronchiectasis neutrophils

We assessed phagocytosis and killing of green fluorescent protein (GFP)*–Pseudomonas* O1 (PAO1), spontaneous neutrophil apoptosis, neutrophil activation (CD62L/CD11b expression) and neutrophil degranulation (myeloperoxidase release). Further information on the specific experiments is provided in the online supplemental file.

### Statistical analysis

Flow cytometry analysis was performed using FlowJo V.10.0.4 (Tree Star, Ashland, Oregon, USA). Results are presented as mean±SEM. Paired and unpaired t-tests were used to compare the two groups, where applicable. Data were analysed by one-way analysis of variance (ANOVA) with Bonferonni’s multiple comparison post hoc test (GraphPad Prism V.6; GraphPad Software, La Jolla, California, USA), when three groups were involved. A repeated measures ANOVA was used where samples from the same participant receive multiple treatments; significance was accepted with p values: *p<0.05.

## Results

Baseline demographics of the participants are shown in table 1.

**Table 1:** Baseline demographics of the study population

Parameters	Patients with bronchiectasis N=34			Healthy volunteers N=6
	Mild n=10	Moderate n=15	Severe n=9	
Age (years)	55 (4.1)	65 (2.2)	64 (2.2)	52 (6.8)
Biological sex (% female)	40	60	22	80
Aetiology				
Idiopathic	10 (100%)	12 (80%)	6 (67%)	
Postinfective		3 (20%)	3 (33%)	
Total WCC (×10^9^/L)	6 (0.5)	6.3 (0.4)	9.3 (1.1)	5.9 (0.5)
Neutrophils	3.3 (0.3)	4.1 (0.3)	6.6 (1.1)	3.5 (0.3)
Eosinophils	0.2 (0.04)	0.3 (0.07)	0.2 (0.06)	0.2 (0.06)
Monocytes	0.5 (0.03)	0.6 (0.05)	0.7 (1)	0.5 (0.05)
ESR (mm/hour)	6.7 (1.8)	13.2 (2.9)	19.6 (6.8)	4.8 (1)
CRP (mg/L)	2.8 (0.5)	4 (1)	16 (7.4)	3.2 (1.1)
FEV_1_ % predicted (L)	95 (5.5)	82 (4)	55 (6.5)	–
FVC % predicted (L)	111 (6)	97 (4)	84 (6)	–
TLCO % predicted (SI)	94% (4.9)	82% (4.2.)	74% (7.8)	–
KCO % predicted (SI)	106% (4.5)	97% (3.7)	100% (7.2)	–
Chronic colonisation	5 (50%)	12 (86%)	8 (89%)	–
Exacerbations in the last year	0.4 (0.3)	2.4 (0.5)	4.2 (0.9)	–
Hospital admissions in the last year	0	0.05 (0.05)	0.7 (0.2)	–

Data presented as mean (±SE of mean).

CRP, C reactive protein; ESR, erythrocyte sedimentation rate; FEV_1_, forced expiratory volume in 1 s; FVC, forced vital capacity; KCO, transfer coefficient corrected for alveolar volume; TLCO, transfer factor for the lung for carbon monoxide; WCC, white cell count.

### Serum lipidomics

LC-MS/MS was done on all serum samples obtained, where available. Only data that passed quality control were used, and thus we have a smaller sample size. The demographics are shown in online supplemental table 1. Samples were divided into mild^9^ and moderate–severe^15^ bronchiectasis groups (moderate and severe groups were combined as lipids were not detected in all samples by lipidomics, and hence data were combined to obtain meaningful interpretation. Baseline demographics of mild^9^ and moderate–severe^15^ groups are provided in online supplemental table T2.

The main lipids assessed were LXA_4_, resolvins, maresins, prostaglandin E_2_ (PGE_2_), 5-hydroxyeicosatetranoic acid (5-HETE), 15-hydroxyeicosatetranoic acid (15-HETE), leukotriene B_4_ (LTB_4_) and 9-hydroxyoctadecadienoic acid (9-HODE). 15-HETE is a precursor of LXA_4_ and is a proinflammatory mediator. LTB_4_ is a proinflammatory cytokine and 9-HODE is a proinflammatory metabolite produced from arachidonic acid.

There were significantly higher levels of PGE_2_, 15-HETE and LTB_4_ in patients with moderate–severe bronchiectasis compared with healthy controls (p=0.03, p=0.03 and p=0.02, respectively). Although there was a trend towards higher 5-HETE and 9-HODE levels in moderate–severe bronchiectasis compared with healthy volunteers, this failed to reach statistical significance (p=0.3 and 0.2, respectively). Although there was a trend towards higher PGE_2_, 15-HETE, LTB_4_, 5-HETE and 9-HODE levels between the mild bronchiectasis and healthy volunteers, this was not statistically significant (figure 1). LXA_4_, resolvins and maresins were not detected in any of the samples by LC-MS/MS.

### Blood LXA_4_ measured by ELISA

**Figure 2:**
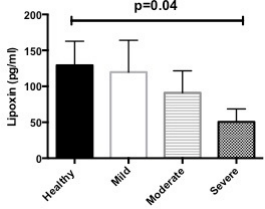
Significantly lower level of LXA_4_ is severe bronchiectasis. Healthy, n=6; mild, n=10; moderate, n=15; severe, n=9. Pooled data represented as mean±SEM. LXA_4_, lipoxin A_4_.

LXA_4_ was detected by ELISA. Using one-way ANOVA, we found that there was a significantly lower level of LXA_4_ in severe bronchiectasis, p=0.04 (figure 2), as measured by ELISA.

### BALF lipidomics

**Figure 3:**
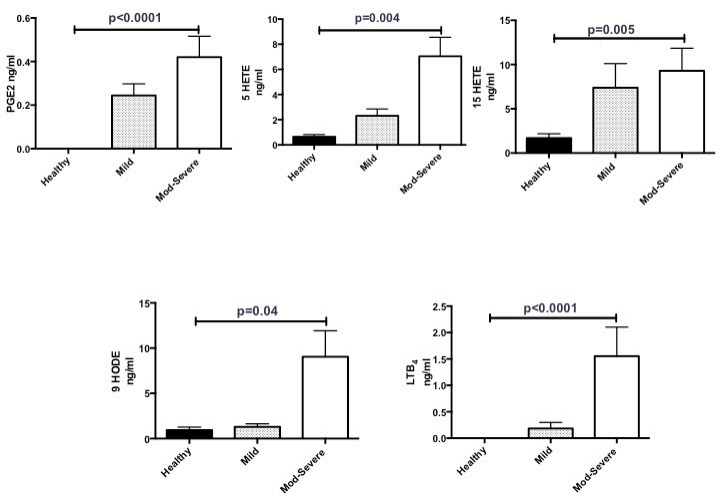
Significantly higher levels of PGE_2_, 5-HETE, 15-HETE, 9-HODE and LTB_4_ detected in patients with moderate–severe disease compared with healthy volunteers. Lipidomics obtained by mass spectrometry and liquid chromatography. pooled data presented as mean±SEM. One-way analysis of variance used for comparisons. Healthy=6 volunteers, mild= 9 patients, moderate–severe=15 patients. 5-HETE, 5-hydroxyeicosatetranoic acid; 15-HETE, 15-hydroxyeicosatetranoic acid; 9-HODE, 9-hydroxyoctadecadienoic acid; LTB_4_, leukotriene B_4_; PGE_2_, prostaglandin E_2_.

There were significantly higher levels of PGE_2_, 5-HETE, 15-HETE, 9-HODE and LTB_4_ in patients with moderate–severe bronchiectasis compared with healthy volunteers (p<0.0001, p=0.004, p=0.005, p=0.04 and p<0.0001, respectively) (figure 3). Although there was a trend, there was no statistically significant difference in the PGE_2_, 15-HETE, LTB_4_, 5-HETE and 9-HODE levels between mild bronchiectasis and healthy volunteers. LXA_4_, resolvins and maresins were not detected in any of the samples by LC-MS/MS. PGE_2_ was not detectable in BALF from healthy individuals.

### Correlation of blood PGE_2_ and LTB_4_ to markers of disease severity

**Figure 4:**
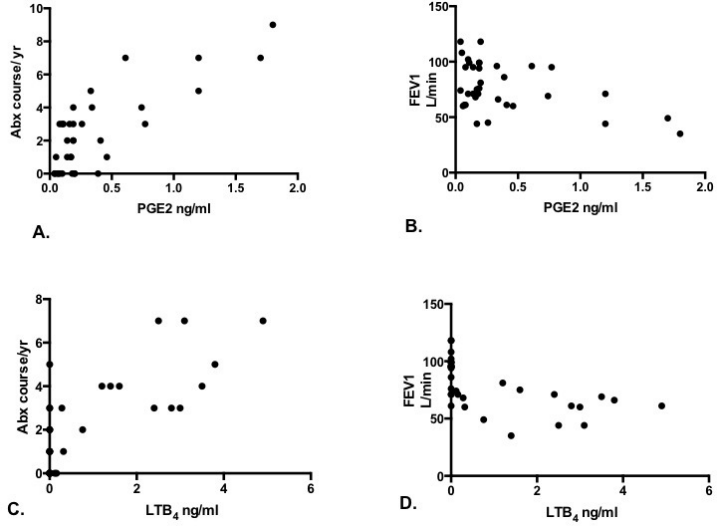
Using Pearson two-tailed correlation coefficient, we found that there was a correlation between serum PGE_2_ and antibiotic courses received in the preceding year and % predicted FEV_1_ (A, B). Also, there was a correlation between serum LTB_4_ and antibiotic courses received in the preceding year and % predicted FEV_1_ (C, D). FEV_1_, forced expiratory volume in 1 s; LTB_4_, leukotriene B_4_; PGE_2_, prostaglandin E_2_.

There was a positive correlation of PGE_2_ with antibiotic courses (r=0.78, 95% CI 0.61 to 0.88, p<0.0001) and a negative correlation with % predicted forced expiratory volume in 1 s (FEV_1_) (r=−0.46, 95% CI −0.15 to −0.69, p=0.004). Similarly, there was a positive correlation of LTB4 with antibiotic courses (r=0.76, 95% CI 0.56 to 0.87, p<0.0001) and a negative correlation with % predicted FEV_1_ (r=−0.58, 95% CI −0.3 to −0.76, p=0.0003; figure 4). There was no correlation of PGE_2_ or LTB_4_ to other parameters of the disease severity on the BSI. There was no correlation of 15-HETE, 5-HETE and 9-HODE to markers of disease severity.

### Effect of biological sex on PGE_2_

There was biological sex imbalance in our cohort, and to investigate this further, there was a subanalysis of blood PGE_2_. The blood PGE_2_ was 1.1 (±0.3) for male healthy volunteers and 1.3 (±0.5) for female healthy volunteers (p=0.2), 4.2 (±1.4) for male mild bronchiectasis and 3.9 (±0.1) for female mild bronchiectasis (p=0.8), 2.9 (±0.6) for male moderate–severe bronchiectasis, and 4.2 (±0.7) for female moderate–severe bronchiectasis (p=0.2). This is a small study and so detailed further subanalysis was not carried out.

### Effect of LXA_4_ on blood neutrophil function

**Figure 5:**
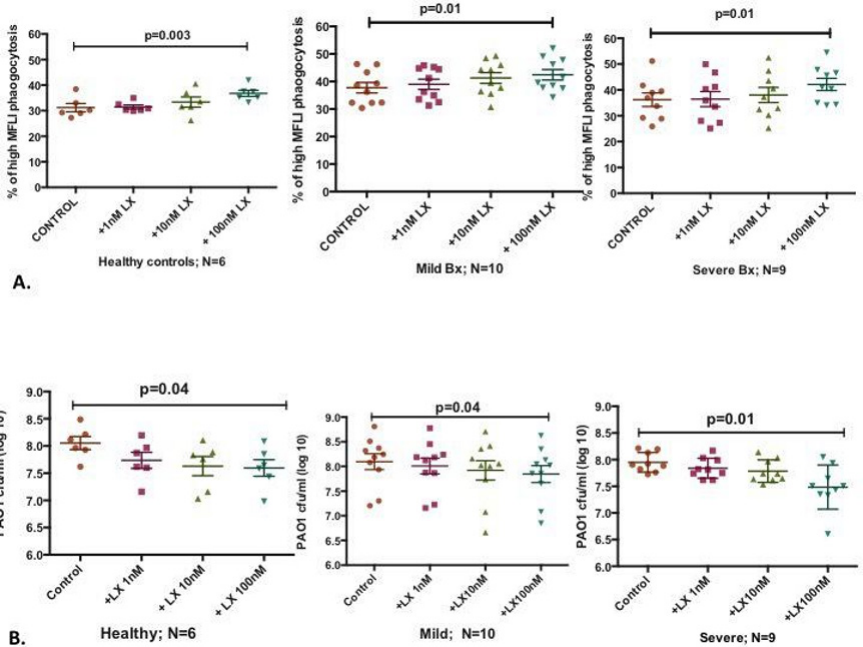
(A) There was a concentration-dependent increase in phagocytosis induced by LXA_4_, in healthy volunteers, mild and severe bronchiectasis. One-way ANOVA with a Bonferroni correction for multiple comparisons, with p values representing the comparison of control to 1, 10 and 100 nM of LXA_4_. Pooled % neutrophil phagocytosis data, showing means±SEM. (B). There was dose-dependent increase in killing with LXA_4_ in healthy volunteers, mild and severe bronchiectasis. One-way ANOVA with Bonferonni’s correction for multiple comparisons used, with p values representing the comparison of control to 1, 10 and 100 nM of LXA_4_. Pooled % neutrophil killing data showing means±SEM. ANOVA, analysis of variance; LXA_4_, lipoxin A_4_.

As we detected significantly lower levels of LXA_4_ in blood of patients with severe bronchiectasis, we investigated the effect of LXA_4_ on reprogrammed neutrophil function.^15^ With the complexity of the experiments, the authors studied healthy controls and patients with mild and severe bronchiectasis only. Blood neutrophils from healthy volunteers (n=6) and patients with mild (n=10) and severe bronchiectasis (n=9) were pretreated ex vivo with varying concentrations of LXA_4_ for 30 min and then coincubated with GFP–PAO1. Phagocytosis of bacteria (GFP–PAO1) was assessed after 15 min and killing after 24 hours. Total phagocytosis was calculated by assessing neutrophils positive for GFP. Data were analysed by gating the overall phagocytosis first. There was no difference in overall phagocytosis in healthy controls and in patients with mild and severe bronchiectasis. Next, we gated the neutrophils that had taken up much higher number of bacteria, as indicated by the mean fluorescence (fluorescence intensity, FLI). This was done at 50% of the total phagocytosis and we called this high MFLI phagocytosis. In healthy controls, mild and severe bronchiectasis, LXA_4_ was able to significantly improve phagocytosis (comparing only the high MFLI and not total phagocytosis) (figure 5A) and killing of GFP–PAO1 in a concentration-dependent manner with statistical significance achieved at 100 nM LXA_4_ (figure 5B).

### Effect of LXA_4_ on airway neutrophil function

Airway neutrophils were isolated from BALF from patients with bronchiectasis (no airway neutrophils could be isolated from healthy volunteers), and phagocytosis and killing assays were performed using GFP–PAO1.

**Figure 6:**
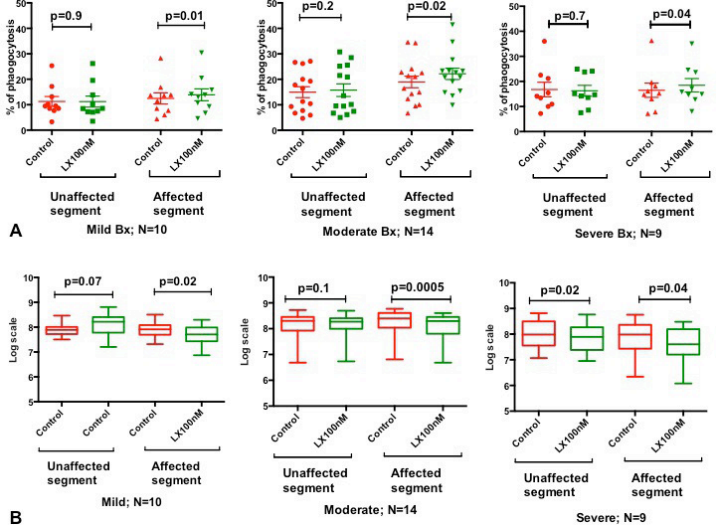
(A) Bacterial phagocytosis and (B) killing. LXA_4_ significantly increased phagocytosis and killing of PAO1 by airway neutrophils isolated from the lung segments affected by Bx in mild, moderate and severe diseases. In unaffected segments, LXA_4_ only had an improvement in bacterial killing in patients with severe Bx only. Pooled data presented as mean±SEM. Paired t-tests used for all comparisons. Bx, bronchiectasis; LXA_4_, lipoxin A_4_; PAO1, *Pseudomonas* O1.

Neutrophils collected from affected lung segments demonstrated statistically significant improvement in phagocytosis (neutrophil gating done as in previous experiment) and killing after treatment with 100 nM LXA_4_ in mild, moderate and severe bronchiectases (figure 6A, B). The neutrophils collected from unaffected lung segments displayed significant improvement in bacterial killing after treatment with 100 nM LXA_4_ in patients with severe bronchiectasis only (p=0.02).

### Effect of LXA_4_ on stable disease-state peripheral blood neutrophil spontaneous apoptosis, surface expression of CD11b and CD62L, and myeloperoxidase release

For this experiment, blood neutrophils from healthy volunteers and patients with stable-state bronchiectasis with either mild and moderate–severe disease were pretreated with LXA_4_ 1, 10 and 100 nM and assessed for the onset of spontaneous apoptosis. LXA_4_ did not significantly modulate spontaneous apoptosis or viability of neutrophils (p=0.4, p=0.5 and p=0.4, respectively), in contrast to roscovitine, used as a positive control for apoptosis induction (online supplemental figure S2).

To evaluate the effect of LXA_4_ on fMLF-induced neutrophil activation, surface expression of CD11b and CD62L was measured. In this experiment, LXA_4_ treatment induced a small but statistically significant reduction in fMLF-induced upregulation of CD11b and shedding of CD62L in a concentration-dependent manner, in healthy volunteers and in patients with mild and moderate–severe bronchiectases (online supplemental figure S3 and 4).

Additionally, LXA_4_ reduced neutrophil degranulation and release of myeloperoxidase in a concentration-dependent manner from neutrophils isolated from healthy volunteers and in patients with mild and moderate–severe bronchiectasis (online supplemental figure S5).

## Discussion

This is the first study characterising the lipid profile in bronchiectasis blood and airways in the stable state. We established that there is a dysregulation of the lipids in serum and airways in the stable state. In serum, there was significantly higher levels of the proinflammatory metabolites PGE_2_, 15-HETE and LTB_4_ and significantly lower levels of the anti-inflammatory mediator LXA_4_ in severe disease compared with mild disease and healthy volunteers. Although LXA_4_ was not detectable in serum using LC-MS/MS, it was detected by ELISA. This can be explained by level of sensitivity of the assays used. Detection limits were in nanogram per millilitre by LC-MS and in picogram per millilitre by ELISA. In the airways, there were significantly higher levels of PGE_2_, 5-HETE, 15-HETE, 9-HODE and LTB_4_. There was a correlation of PGE_2_ and LTB_4_ with antibiotic courses and % predicted FEV_1_. Higher levels of PGE_2_ and LTB_4_ were inversely related to the % predicted FEV_1_ and directly related to the number of antibiotic courses for bronchiectasis exacerbations.

In the stable state, LXA_4_ was able to significantly improve phagocytosis and killing of GFP *Pseudomonas aeruginosa* by blood and airway neutrophils, in a concentration-dependent manner. In addition, the authors demonstrated that LXA_4_ reduced fMLF-induced neutrophil activation. However, there was no effect of LXA_4_ on spontaneous neutrophil apoptosis.

To the authors’ best knowledge, prostaglandins have not been studied in bronchiectasis. In COPD, PGE_2_ levels are increased in the exhaled breath and are known to correlate with airflow obstruction.^16 17^ Additionally, studies have demonstrated that PGE_2_ is a critical component in amplifying and perpetuating senescence and inflammation in COPD fibroblasts.^18^ PGE_2_ enhances LTB_4_-mediated polymorphonuclear leucocyte extravasation and tissue injury that is blocked by topical administration of synthetic LXA_4_.^19^ However, prostaglandins are also key in the temporal switch of LTB_4_ to LXA_4_—a term coined as ‘lipid mediator class switching’.^20^ In the stable state, the authors demonstrated that PGE_2_ and LTB_4_ were significantly higher and LXA_4_ was significantly lower in blood in severe bronchiectasis. Serum PGE_2_ and LTB_4_ levels were correlated to airflow obstruction as measured by FEV_1_. Higher serum PGE_2_ and LTB_4_ levels were also correlated to more exacerbations requiring antibiotic courses in bronchiectasis, both markers of disease severity in bronchiectasis.^12^ Studies have established that disease severity in bronchiectasis predicts mortality, hospital admissions, exacerbations, quality of life, respiratory symptoms, exercise capacity and lung function decline in bronchiectasis.^21^ This dysregulation between lipid mediators that the authors have shown here would thereby lead to more inflammation even in the stable state. Although PGE_2_ levels are known to initiate the class switching during resolution of inflammation, this was not demonstrated in our study. Almost certainly, the levels of LXA_4_ detected in bronchiectasis serum are unable to counter-regulate the production of the proinflammatory LTB_4_.

In addition to PGE_2_ and LTB_4_, the other metabolites (5-HETE, 15-HETE and 9-HODE) detected in our study are also proinflammatory mediators, and they remain elevated even in the stable state in moderate and severe bronchiectases compared with mild disease. LTB_4_ has been studied in bronchiectasis and is known to be one of the major chemotactic factors in the bronchial airways in bronchiectasis.^22^ Additionally, there is evidence to suggest that LTB_4_ is raised during an exacerbation and reduces with antibiotic therapy.^23^ Persistently elevated lipid mediators in bronchiectasis, in part, may explain why more patients with moderate–severe bronchiectasis have a higher mortality rate and more hospital admissions compared with patients with mild disease.^12^

LXA_4_ was able to improve blood and airway neutrophil bacterial phagocytosis and killing, thereby enhancing bacterial clearance and potentially having long-term consequences on infection, inflammation and resolution. The impact of LXA_4_ on bronchiectasis airway neutrophil function was more pronounced when neutrophils were isolated from a disease-affected lung region than an unaffected region. The mechanism underpinning this observation remains to be determined, but this raises the intriguing possibility that regional lung patterning of LXA_4_ deficiency might exist, with cells collected having had divergent in vivo exposure to LXA_4_ before experimental use.

LXA_4_ was able to reduce fMLF-induced CD11b upregulation, CD62L shedding and myeloperoxidase release. These anti-inflammatory functions of LXA_4_ have previously been demonstrated in the literature.^17^ However, this is the first time that the effects of lipoxins have been demonstrated on a subset of reprogrammed neutrophils from patients with bronchiectasis.

Biological sex may have an effect on the dynamics of the lipids in bronchiectatic airways. We did not identify differences with blood PGE_2_, but as this was a small study, further detailed subanalysis was not carried out. Further studies would be needed to explore this.

The authors have shown that serum neutrophils are reprogrammed in bronchiectasis, leading to persistent and unresolving inflammation.^15^ This study now demonstrates that there is a dysregulation of the lipid mediators and failure of class switching during inflammation in bronchiectasis, despite adequate levels of PGE_2_. Whether there is a role of cyclo-oxygenase inhibitors in bronchiectasis to block the production of PGE_2_ needs to be explored further. Certainly, with the emergence of antibiotic resistance, the role of novel specialised proresolving lipid mediators is promising, especially in bronchiectasis, where recurrent exacerbations requiring antibiotic therapy is one of the cardinal features of the disease.

### Limitations of the study

There are a couple of limitations in this study: first, that this is a small study, and second, this study did not assess the role LXA_4_ on bronchiectasis neutrophils during exacerbations.

## Conclusion

There is a dysregulation of lipid mediators in bronchiectasis in the stable state with excess proinflammatory lipids. LXA_4_ improves the function of reprogrammed neutrophils. The therapeutic efficacy of LXA_4_ in bronchiectasis warrants further studies.

## Data Availability

All data relevant to the study are included in the article or uploaded as supplementary information. Relevant data is included in the manuscript.
