# Supplementary material for: Dysregulation of prostaglandins, leukotrienes and lipoxin A4 in bronchiectasis
Source: Thorax. 2021 Nov 17;77(10):960–7. doi: 10.1136/thoraxjnl-2020-216475 (PMC9510413; doi:10.1136/thoraxjnl-2020-216475)
Supplement: Supplementary data [file thoraxjnl-2020-216475supp001.pdf]

On line supplement

Solid Phase Extraction (SPE) Chromatography

ISOLUTE C18 500 mg/6 mL cartridges were conditioned with 3x 6 mL methanol and 2x 6 mL HPLC grade water. Samples were applied and allowed to run through by force of gravity. Afterwards cartridges were first washed with half the sample volume of HPLC grade water and then with 2x 6 mL hexane. Compounds were eluted with 6 mL ethylacetate and dried under vacuum. Samples were re-dissolved in 100 µL methanol/water 50/50 and stored at -80°C until analysis.

Liquid Chromatography-Tandem Mass Spectrometry (LC-MS/MS) Analysis

Samples were analysed on a Thermo Scientific TSQ Quantum Ultra triple quadrupole mass spectrometer system coupled to a Thermo Scientific Accela 1250 pump and autosampler. Separation was achieved on a Phenomenex Kinetex XB-C18 LC column of 100 x 2.1 mm with 1.7 µm particle size, maintained at 32°C. The injection volume was 10 µL. A solvent gradient was run with solvent A consisting of 90% water, 10% methanol and 0.1% acetic acid and solvent B consisting of 100% methanol and 0.1% acetic acid. The gradient was the following: linear increase from 45 to 60% solvent B between 0 and 10 minutes, linear increase to 70% solvent B between 10 and 11 minutes, linear increase to 100% solvent B between 11 and 18 minutes, holding at 100% solvent B between 18 and 20 minutes, decrease back to 45% solvent B between 20 and 21 minutes and column equilibration at 45 % solvent B between 21 and 25 minutes.

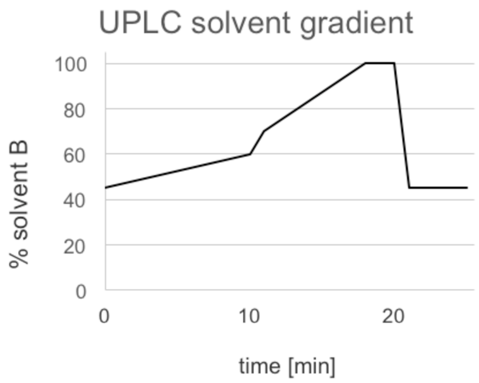

Figure: S1. Graph of a representative sample.

The mass spectrometer was coupled to an electrospray ionisation (ESI) source operated in negative mode and compounds were detected by multiple reaction monitoring (MRM). 56 compounds were monitored, including six deuterium labelled internal standards (see table 6 below, internal standards shown in italics). Compounds were quantified through comparison to original standards. The LC-MS/MS system was controlled and data analysis was performed with Thermo Xcalibur 2.2 software. Results were normalised to the sample volume and recovery of the associated internal standard.

Compound	Precursor ion	Product ion	Compound	Precursor ion	Product ion
**6 keto PGF1a**	369.2	163.2	9 HETE	319.2	151.3
**20 OH LTB_4**	351.2	195.2	11,12 dHET	337.2	167.2
**20 COOH LTB_4**	365.2	195.1	*14,15 dHET d11*	*348.3*	*207.2*
**11 dehydro TxB_2**	367.2	161.2	15 HEPE	317.2	219.2
**8IsoPGF2a**	353.2	193.2	8,9 dHET	337.2	127.2
**TxB2**	369.2	169.1	12 HEPE	317.2	179.2
**iPF2a VI**	353.2	115.2	5 HEPE	317.2	115.2
**11beta PGF2a**	353.2	193.1	5,6 dHET	337.2	145.1
**PGE2**	351.2	271.2	13 HODE	295.3	195.2
**dihydro PGE2**	351.2	235.0	20 HETE	319.2	245.3
PGE2 d4	*355.2*	*193.2*	7 HDHA	343.2	141.2
**PGD2**	351.2	233.1	9 HODE	295.3	171.2
**dihydro PGD2**	351.2	175.2	15 OxoETE	317.2	113.3
**PGF2a**	353.3	193.2	15 HETE	319.2	219.2
**RvD2**	375.2	175.1	8 HETE	319.2	155.2
**LXA_4**	351.2	115.3	11 HETE	319.2	167.2
**RvD1**	375.2	215.2	12 HETE	319.3	179.2
**LTD_4**	495.3	177.0	*15 HETE d8*	*327.3*	*226.1*
**5,6 EET**	319.2	191.3	17 HDHA	343.3	281.3
**Maresin 1**	359.2	177.2	14 HDHA	343.3	205.2
**LTB_4**	335.2	195.2	9,10 EpOME	295.2	171.2
**LTB_4 d4**	*339.2*	*197.2*	5 OxoETE	317.2	203.3
**10,17 diHDHA**	359.2	153.2	5 HETE	319.3	115.2
**LTE4**	438.2	333.3	*14,15 EET d11*	*330.3*	*175.2*
LTE4 d5	*443.3*	*338.2*	11,12 EET	319.2	167.2
**15deoxy D 12,14 PGJ2**	315.2	271.2	8,9 EET	319.3	155.2
**14,15 dHET**	337.2	207.2	14,15 EET	319.3	175.2
**9,10 diHOME**	313.2	201.2			

Table T1: Compounds monitored by liquid chromatography mass spectrometry.

Effect of LXA_4 on stable disease state peripheral blood neutrophil spontaneous apoptosis, surface expression of CD11b and CD62L and myeloperoxidase release

For this experiment, blood neutrophils from healthy volunteers, and stable state bronchiectasis patients with either mild or severe disease were pre-treated with LXA_4 1nM, 10nM, and 100nM and assessed for the onset of spontaneous apoptosis. LXA_4 did not significantly modulate spontaneous apoptosis or viability of neutrophils (p=0.4, p=0.5 and p=0.4 respectively), in contrast to Roscovitine, used a positive control for apoptosis induction (figure S1).

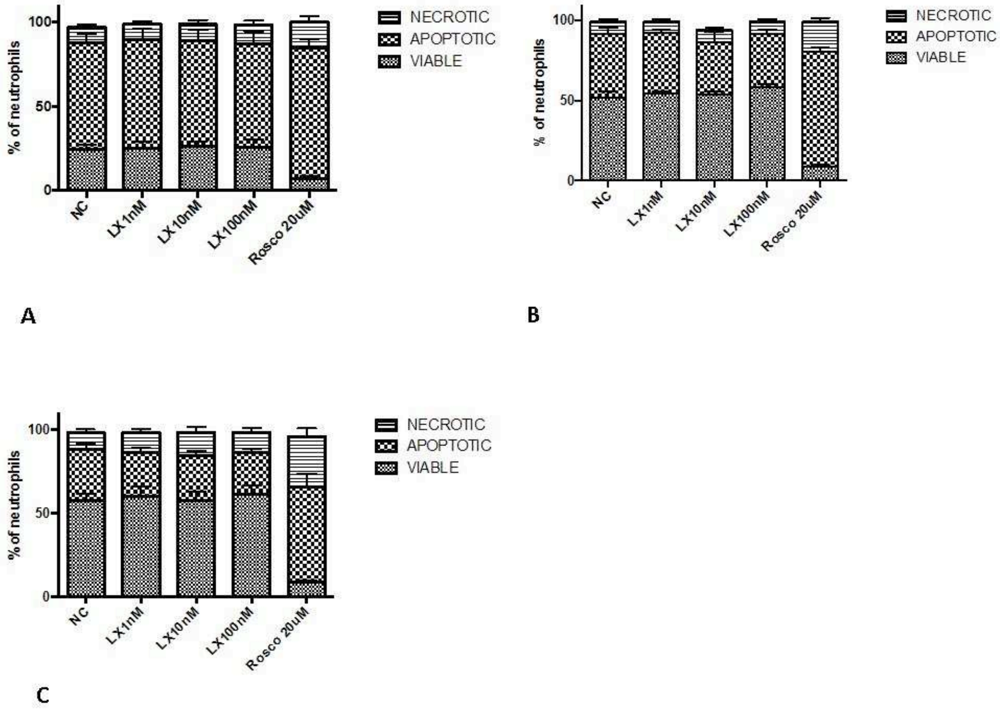

Figure S2. Blood neutrophils from (a) healthy volunteer, (b) mild and (c) severe bronchiectasis patients were cultured for 20 hours and cell viability, apoptosis and necrosis were assessed by flow cytometry. There was no effect of LXA_4, even at a maximum dose of 100nM, on spontaneous apoptosis at 20 hours. Pooled data presented as mean +/- SEM; n=6 per group. Analysis by two way ANOVA. Roscovitine is a CDK-inhibitor that promotes apoptosis and was used as a positive control (5).
LX= Lipoxin A_4; NC= negative control; rosco= roscovitine.

Effect of LXA_4 on fMLF induced upregulation of CD11b

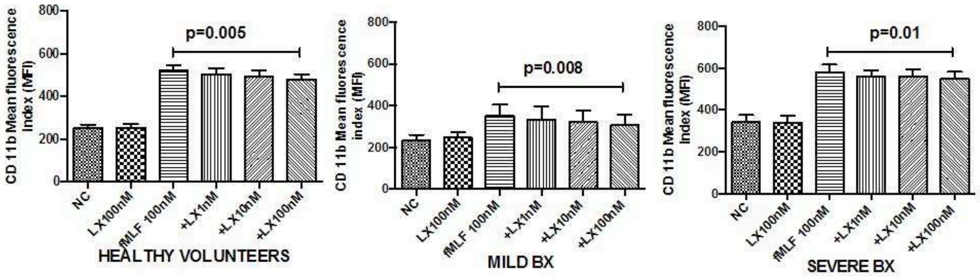

Figure S3. There was a concentration-dependent reduction of fMLF-induced activation (30 minutes) and CD11b expression by LXA_4; p=0.005 healthy volunteers; p=0.008 mild bronchiectasis; p=0.01 severe bronchiectasis. Pooled data presented as mean ± SEM; n=6 per group. Analysis by one-way ANOVA. BX= bronchiectasis; NC= negative control; fMLF= n- formyl-methyl- leucyl- phenylalanine; LX= Lipoxin A_4.

Effect of LXA_4 on fMLF induced shedding of CD62L

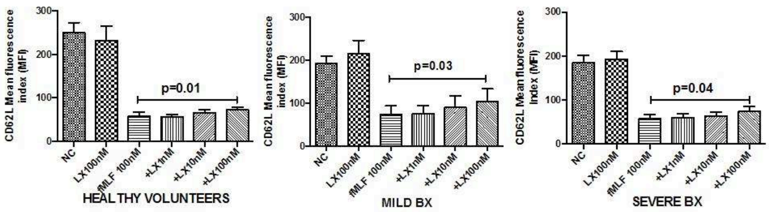

Figure S4. There was a concentration-dependent reduction of fMLF-induced activation and CD62L shedding by LXA_4; p=0.01 healthy volunteers; p=0.03 mild bronchiectasis; p=0.04 severe bronchiectasis. Pooled data presented as mean ± SEM; n=6 per group. Analysis was done by one-way ANOVA. BX= bronchiectasis; NC= negative control; fMLF= n- formyl-methyl- leucyl- phenylalanine; LX= Lipoxin A_4.

Effect of LXA_4 of neutrophil degranulation and myeloperoxidase release

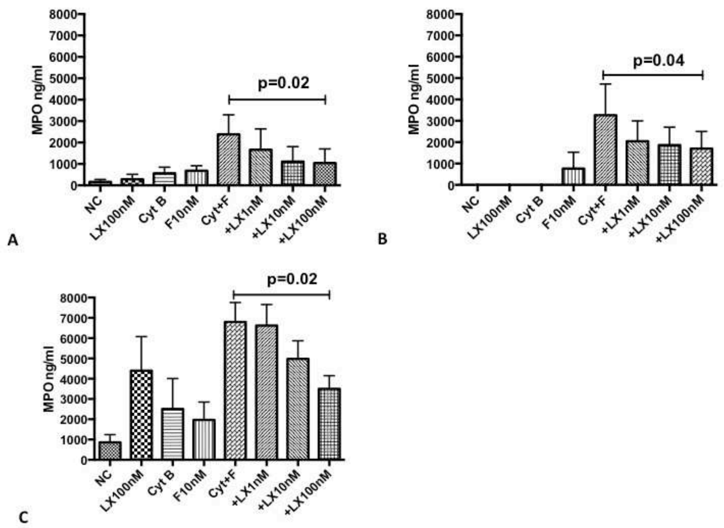

Figure S5. There was a concentration-dependent reduction of fMLF (30 minutes) and cytochalasin B (10 minutes) induced activation and release of MPO by LXA_4, (a) p=0.02 healthy volunteers; (b) p=0.04 mild bronchiectasis; (c) p=0.02 severe bronchiectasis (In severe bronchiectasis, LX 100nM on its own seems to increase MPO generation - this is due to one experimental data set, which skewed the overall result). Pooled data presented as mean ± SEM. Analysis done by one way ANOVA. Cyt= Cytochalasin B; F=fMLF= n-formyl- methyl-leucyl-phenylalanine; NC= negative control; LX= LXA_4.

Parameters	Mild (N=9)	Moderate-Severe (N=15)
**Age**	55 (5)	68 (2.2)
**Gender (% female)**	40%	53%
**Aetiology**<br>**Idiopathic**<br>**Post infective**	 9 (100%)<br>0	 11 (74%)<br>4 (26%)
**Total WCC (x10^9/L)**	6.3 (0.8)	7 (0.5)
**Neutrophils**	3.3 (1)	4.4 (0.4)
**Eosinophils**	0.2 (0.09)	0.2 (0.03)
**Monocytes**	0.5 (0.06)	0.6 (0.07)
**ESR (mm/hr)**	6 (2.1)	17 (4.4)
**CRP (mg/L)**	2.2 (0.5)	10 (4)
**FEV_1% predicted**	101 (6)	73 (5.6)
**FVC % predicted**	117 (1.5)	95 (4.6)
**TLCO % predicted**	100 (7)	80 (5)
**KCO % predicted**	104 (6)	97 (4)
**Chronic colonisation**	2 (22%)	12 (80%)
**Exacerbations in the last year**	0.2 (0.2)	3.6 (0.6)
**Hospital admissions in the last year**	0	0.3 (0.1)

Table T2. Baseline demographics of the sub study population. Data presented as mean (± standard error of mean).

CRP= c reactive protein; ESR= erythrocyte sedimentation rate; FEV_1= forced expiratory volume in 1sec; FVC= forced vital capacity; KCO= transfer coefficient corrected for alveolar volume; TLCO= transfer factor for the lung for carbon monoxide; WCC= white cell count.
